# Adipose tissues and thyroid hormones

**DOI:** 10.3389/fphys.2014.00479

**Published:** 2014-12-11

**Authors:** Maria-Jesus Obregon

**Affiliations:** Department of Molecular Physiopathology, Instituto de Investigaciones Biomedicas “Alberto Sols” (IIBM), Consejo Superior de Investigaciones Cientificas and Universidad Autonoma de MadridMadrid, Spain

**Keywords:** BAT, thermogenesis, adipogenesis, lipogenesis, lipolysis, deiodinases, brite adipocytes, “browning”

## Abstract

The maintenance of energy balance is regulated by complex homeostatic mechanisms, including those emanating from adipose tissue. The main function of the adipose tissue is to store the excess of metabolic energy in the form of fat. The energy stored as fat can be mobilized during periods of energy deprivation (hunger, fasting, diseases). The adipose tissue has also a homeostatic role regulating energy balance and functioning as endocrine organ that secretes substances that control body homeostasis. Two adipose tissues have been identified: white and brown adipose tissues (WAT and BAT) with different phenotype, function and regulation. WAT stores energy, while BAT dissipates energy as heat. Brown and white adipocytes have different ontogenetic origin and lineage and specific markers of WAT and BAT have been identified. “Brite” or beige adipose tissue has been identified in WAT with some properties of BAT. Thyroid hormones exert pleiotropic actions, regulating the differentiation process in many tissues including the adipose tissue. Adipogenesis gives raise to mature adipocytes and is regulated by several transcription factors (c/EBPs, PPARs) that coordinately activate specific genes, resulting in the adipocyte phenotype. T3 regulates several genes involved in lipid mobilization and storage and in thermogenesis. Both WAT and BAT are targets of thyroid hormones, which regulate genes crucial for their proper function: lipogenesis, lipolysis, thermogenesis, mitochondrial function, transcription factors, the availability of nutrients. T3 acts directly through specific TREs in the gene promoters, regulating transcription factors. The deiodinases D3, D2, and D1 regulate the availability of T3. D3 is activated during proliferation, while D2 is linked to the adipocyte differentiation program, providing T3 needed for lipogenesis and thermogenesis. We examine the differences between BAT, WAT and brite/beige adipocytes and the process that lead to activation of UCP1 in WAT and the presence of BAT in humans and its relevance.

## Introduction

Thyroid hormones regulate multiple physiological systems in many tissues and are of maximal importance during developmental processes. T3 regulates the development of many tissues (Bernal, 2002; Morreale De Escobar et al., 2004) by acting in specific cells, for example in the cochlea or the retina (Forrest et al., 1996; Roberts et al., 2006). The supply of thyroid hormones is finely tuned and regulated in a time- and dose-specific way in specific areas of the brain, through sequential increases or decreases in D2 and D3 deiodinases, as studied in human fetal brain (Kester et al., 2004), in the cochlea (Ng et al., 2004) or during the metamorphosis of amphibians and fishes (Brown et al., 1996; Isorna et al., 2009). Thyroid hormones act by regulating genes involved in the differentiation program of many tissues. During adult life thyroid hormones regulate the function of many tissues, as brain, muscle, heart, liver, adipose tissue or skin by controlling the metabolism of carbohydrates, lipids, the transcription of many proteins (Mullur et al., 2014) and the basal metabolic rate. T3 acts through their nuclear receptors, which are encoded by two genes TRα and TRβ, with different isoforms: TRα-1, TRα-2, and TRβ-1. They bind to thyroid response elements (TREs) present in the promoters of the target genes, forming heterodimers with RXR. T3 actions are modulated by corepressors and coactivators. Thyroid hormone concentrations are modulated in tissues by the action of the deiodinases D1, D2, and D3 that control the amount required in each tissue.

The adipose tissue is one important target of thyroid hormones. The adipose tissue is the main place for lipid storage, besides its function in lipid transport, synthesis and mobilization. The adipose tissue stores energy in the form of fat, so that this metabolic energy can be used in times of hunger or illness. In addition, adipose tissue works as a homeostatic mechanism regulating energy reserves and releasing many substances that keep the homeostasis of the organism, some of them, like leptin, act as adipostats regulating the amount of fat stored.

Mammals have two types of adipose tissue: white and brown adipose tissue (WAT and BAT), with different phenotype, function and regulation. The white adipose tissue (WAT) was considered for many years a site for lipid storage. White adipocytes have a characteristic large lipid droplet that fills the cellular space, while the cellular structures (nuclei, mitochondria) are placed near the cellular membrane. WAT is distributed in different anatomical locations that have been grossly divided into subcutaneous and visceral fat o intra-abdominal fat. Both locations have different lipolytic sensitivity in response to hormones and its abundance is associated to a different health risk, because an increase in visceral fat is associated to insulin resistance, metabolic syndrome and cardiovascular diseases (Wajchenberg, 2000). Other organs as the kidney, heart and the gonads (perirenal or perigonadal depots) are also cover by fat. Those adipose locations are not pure WAT and some of them are located in the primitive BAT locations as found in hibernating animals. In humans WAT is one of the largest tissues and is found in many depots all along the body, it accounts for about 10–15% of the total body weight in control subjects, and this percentage increases up to 50% in obese subjects.

The brown adipose tissue (BAT) is responsible for the adaptive or facultative thermogenesis. BAT is activated in response to cold exposure or fat diets providing extra heat in demanding situations to maintain energy balance. BAT is abundant in small rodents, hibernating animals and specially in newborns (Cannon and Nedergaard, 2004); it is found in small pads in the interscapular and cervical region, protecting organs as the heart, aorta, kidneys and other organs that should be heated up during the arousal from hibernation. The main function of BAT is to produce heat. This is possible by the activation of the uncoupling protein 1 (UCP1), a mitochondrial protein that acts as proton channel, uncoupling the oxidative phosphorylation and producing heat, instead of ATP. This activation is switch on by the adrenergic stimulation that increases after cold exposure. BAT is a highly innervated and irrigated tissue. BAT morphology is characterized by multilocular lipid droplets that can be easily mobilized and multiple and active mitochondria which number and activity increases under cold exposure (mitochondriogenesis). Today, the activation of BAT is considered as a possible therapeutic tool to fight obesity.

**Figure 1:**
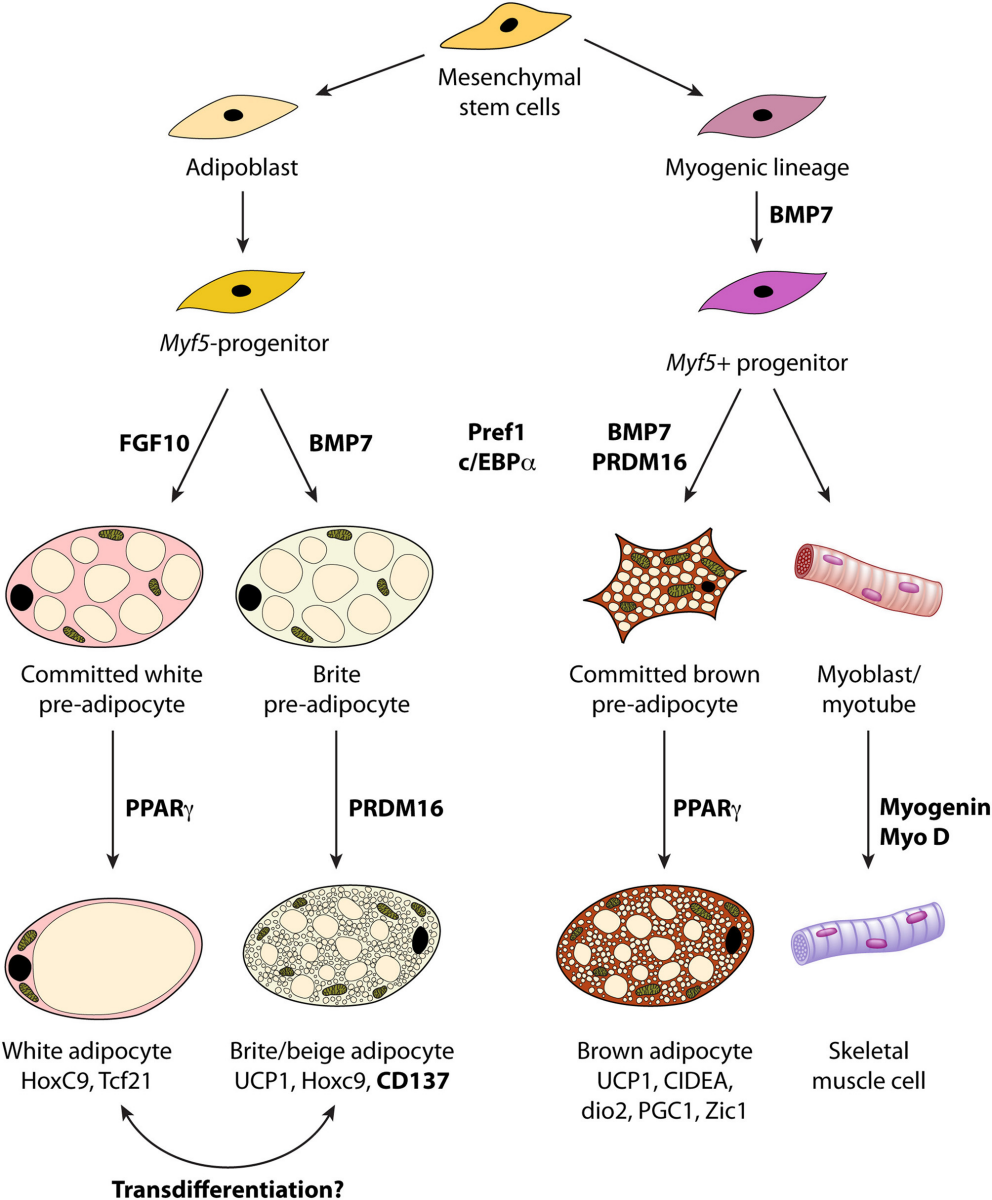
**Lineage of white, brown and “brite/beige” adipocytes from stem cells and molecular markers involved in the developmental program**. BMP, bone morphogenetic protein; UCP1 uncoupling protein 1; PGC1, Peroxisome proliferator-activated receptor gamma coactivator 1.

The analysis of the lineage of white and brown adipocytes reveals that both cells have a distinct embryological origin. Brown adipocytes have a myogenic origin, different from white adipocytes, defined by the expression of the myogenic marker, myogenic factor 5, Myf5+ that is also found in myoblasts (Gesta et al., 2007; Timmons et al., 2007). Several genes have been identified to trace the presence of white and brown adipocytes (see below and Figure 1), as well as markers of its terminal differentiation: UCP1 and D2.

In addition another type of adipose tissue has been identified recently identified, called “beige” or “brite” adipose tissue. Under certain circumstances WAT contains small clusters of brown-like adipocytes that express UCP-1, which have been called “brite” (brown-white) or beige adipocytes. They are multilocular and express UCP-1, Cidea (Cell death activator CIDE-A) and other markers of brown adipocytes as PGC1α (PPARγ Coactivator 1α). They are more frequently in certain anatomical locations, e.g., the inguinal fat. “Brite” adipocytes seem to come from different embryonic precursors than brown adipocytes and express distinct gene signatures (Petrovic et al., 2010). Its presence, abundance and increase in activity are regulated different than brown adipocytes (Macotela et al., 2012; Walden et al., 2012).

### Proliferation and differentiation of adipocytes. T3 actions

The adipose tissue was often considered a place for lipid storage. Recently it has been a renewed interest on the study of adipose tissue, the adipocyte-specific genes and its regulation, the secretion of adipocytes and the signaling pathways altered in pathological situations as obesity or diabetes. This has lead to a better knowledge of the adipose tissue. The adipocyte is the functional unit of the adipose tissue and is specialized in the storage of lipids. It acquires its full capacity during adipogenesis that involves the proliferation of mesenchymal-type cells and its differentiation that allows the adipocyte to accomplish its specific functions.

The study of adipose tissue was hampered by the lack of good cell culture systems and by the high lipid content of the adipose tissue. The differentiation of the adipocytes was studied in the eighties using preadipose cells lines derived from NIH 3T3 fibroblasts (3T3-L1 and 3T3-F442) (Spiegelman et al., 1983; Lin and Lane, 1994). Several enzymes were identified that increase exponentially during the process of differentiation. Many of them were lipogenic and glycolytic enzymes: the glycerophosphate dehydrogenase (GPD), the lactic dehydrogenase (LDH), the acetyl-CoA carboxylase (ACC), the fatty-acid binding protein aP2, the stearoyl-CoA desaturase (SCD), the fatty acid synthase (FAS) (Mackall et al., 1976; Spiegelman et al., 1983; Bernlohr et al., 1984; Ntambi et al., 1988), the lipoprotein lipase (LPL), malic enzyme (ME), phosphoenolpyruvate carboxykinase (PEPCK) and some new genes as adipsin (Cook et al., 1987; Flier et al., 1989) and adipoQ, nowadays called adiponectin (Hu et al., 1996). There was a progression in the appearance of these proteins, some are expressed as early markers of differentiation and others appear later on (Ailhaud et al., 1992). The transcription factors C/EBPs were identified as one of the earlier molecules necessary for the adipocyte phenotype (Christy et al., 1989, 1991).

The nuclear receptors peroxisome proliferator-activated receptors (PPARs) were identified later on as critical for the adipocyte phenotype, as will be review later on.

#### Proliferation of preadipocytes. Role of T3 and D3

Pluripotential stem cells give rise to preadipocytes, a mesenchymal cell predetermined to be adipocyte. It is not clear which are the specific factors that trigger the transition from pluripotential stem cells into predetermined preadipocytes. A common mesenchymal stem cell produces adipocytes, myoblasts and osteoblasts (Cornelius et al., 1994; Falconi et al., 2007). PPARγ2 activation itself induces the differentiation of mesenchymal cells into adipocytes (Chen et al., 2007), and several HOX genes have been identified as transcription factors that trigger the transition, several HOX genes display specific expression in WAT and 4 HOX genes (HOXA4, HOXB4, HOXC4, HOXD4) discriminate between WAT and BAT (Cantile et al., 2003). Leukemia inhibitory factor (LIF) could be a marker of these initial steps inducing, together with PPARγ2, the adipocyte phenotype (Falconi et al., 2007). The preadipocyte factor 1, Pref-1, also called Dlk1, is an imprinted gene found in preadipocytes and a potent inhibitor of adipogenesis (Swick and Lane, 1992). Pref-1 activates ERK phosphorylation (Smas and Sul, 1993; Kim et al., 2007) and is a marker of proliferation (Figure 1). Using microarrays, Pref-1 was identified as marker of proliferation of brown preadipocytes, while C/EBP and Necdin were expressed during the proliferation of brown as well as white preadipocytes (Timmons et al., 2007). Necdin is an imprinted gene expressed in the paternal allele, that inhibits the activation of PPARγ1 promoter (Macdougald and Burant, 2005).

The preadipocytes are mesenchymal-type cells found in the stroma-vascular fraction (SVF) of the adipose tissue. These precursor cells allowed to set primary cultures of preadipocytes, which proliferate and differentiated in culture (Nechad et al., 1983). Several growth factors present in serum, mainly fibroblast growth factors are mitogenic for brown preadipocytes (Garcia and Obregon, 1997). Cold exposure induces the proliferation of brown preadipocytes as studied *in vivo* using 3H-thymidine, and this proliferation is beta-adrenergic (Bukowiecki et al., 1986; Geloen et al., 1988; Rehnmark and Nedergaard, 1989), while insulin was proposed as a mitotic factor for white adipocytes (Geloen et al., 1989). The increases in DNA synthesis were confirmed in primary cultures of brown preadipocytes using β 1 adrenergic agonists (Bronnikov et al., 1992). In our hands norepinephrine (NE) is a poor mitogen itself, but increases the mitogenic action of serum, growth factors and vasopressin (Garcia and Obregon, 1997) producing true brown adipocytes that express UCP1 (Garcia and Obregon, 2002). Therefore, NE is important for brown adipocyte proliferation, besides its role in thermogenesis increasing UCP1 expression. The fatty acid arachidonic is a good mitogen for brown adipocytes (Garcia et al., 2012). Recent studies have reported the role of activin in the proliferation of white adipocytes (Zaragosi et al., 2010).

Thyroid hormones seem to be anti-mitogenic, as T3 inhibits bFGF and aFGF mitogenic effect in brown preadipocytes (Garcia and Obregon, 2002). Moreover, type III deiodinase (D3) activity and mRNA are strongly induced by growth factors in brown adipocytes (Hernandez and Obregon, 1995; Hernandez et al., 1998) as in other proliferating cells, suggesting the physiological importance of low T3 levels during proliferation. D3 activity and mRNA increases abruptly when serum is added to cultures of brown adipocytes (Hernandez et al., 2007). So, we propose that D3 is a mitogenic marker in brown preadipocytes. On the contrary, D2 activity is low during proliferation, having a role during differentiation, therefore establishing that both deiodinases have an opposite role in BAT.

Few proliferation studies have been done in white preadipocytes, but serum stimulates DNA synthesis and proliferation in white preadipocytes in primary cultures (unpublished results). The specific growth factors governing proliferation of white preadipocytes require further research. White preadipocytes require only T3, insulin and transferrin to proliferate in serum-free medium (Deslex et al., 1987a,b). Moreover, preadipocytes from obese people produce mitogenic factors that induce a higher proliferation rate than those produced by control subjects (Lau et al., 1987). Proteins secreted by macrophages have been proposed to be mitogens in human preadipocytes (Lacasa et al., 2007), but the specific growth factors or adipokines have not been defined although fatty acids have been proposed to be mitogens for adipocytes. FGF10 was proposed as a mitogen for WAT, because in FGF10−/− mouse embryos the development of WAT is greatly impaired, due to a decreased proliferative activity of WAT, indicating that FGF10 and not C/EBPα is required for the proliferation of white preadipocytes (Asaki et al., 2004). Adipose tissue is a source of several growth factors as IGF-I, IGF binding proteins, TNF alpha, angiotensin II, and MCSF that could stimulate proliferation (Hausman et al., 2001, 2008).

#### Differentiation of adipocytes. The role of transcription factors and T3 regulation

The differentiation of adipocyte was first studied in preadipose cells lines (3T3-L1 and 3T3-F442). Differentiation was induced using dexamethasone and IBMX, an agent that increase cAMP levels. T3 was also included in the “differentiation cocktail.” So, we do not know if the effects observed are due to the action of the T3 added or the process of differentiation itself. During adipocyte differentiation the transcription of specific genes and the synthesis of numerous proteins increase, specially the lipogenic enzymes GPD, FAS and ME, and many others as described above (Mackall et al., 1976; Spiegelman et al., 1983). The activation of these proteins and enzymes follows a temporal pattern with different timings for each transcriptional increase. LPL or IGF-1 are early markers, after the transcription factors C/EBPs and PPARγ; those are followed by the lipogenic proteins: FAS, GPD, ME, Glut4, aP2, ACC, the beta-adrenergic receptors and many others. Later markers are PEPCK, the alfa-2 adrenergic receptors, leptin, or adipsin (Ailhaud et al., 1992). Recently, studies using microarrays (Soukas et al., 2001) show that the adipocyte differentiation is different *in vivo* and *in vitro* and is more complex than previously though. Some genes were expressed only in cell lines and others only in cells derived from tissues “*in vivo*” suggesting that some genes were activated only “*in vivo*” to generate the adipocytes.

So, the differentiation of adipocytes is achieved by the coordinate activation of several adipose-specific genes (Rosen and Spiegelman, 2000), regulated by the C/EBPs and PPARs families of transcription factors that are keys for the activation of the genes required for adipogenesis.

The CCAAT/enhancer-binding proteins (C/EBP) belong to the basic leucine zipper family. The C/EBP family (C/EBPα, C/EBPβ, C/EBPδ) recognizes a common DNA-binding element and has tissue-specific expression patterns. C/EBPα is expressed in brown and white adipose tissues, placenta and liver and is a master regulator of adipose tissue development. C/EBPα overexpression induces the differentiation of 3T3-L1 preadipocytes. It works as antimitotic inducing growth arrest (proteins GADD45 and p21) (Mandrup and Lane, 1997). C/EBPα in preadipocytes increases several adipocyte-specific genes (aP2, Glut4) and triglycerides accumulation (Lin and Lane, 1992; Mandrup and Lane, 1997).

C/EBPβ and C/EBPδ are expressed before than C/EBPα and activate C/EBPα, while PPARγ and C/EBPα induce the differentiation into adipocytes. Many genes (SCD1, aP2, S14, PEPCK, Glut4, UCP1, D2) have C/EBP binding domains in their promoters and are activated together during adipogenesis (Christy et al., 1989). During the development of brown adipose tissue during fetal life, C/EBPβ and C/EBPδ increases precede C/EBPα expression (Manchado et al., 1994).

Mice with a deletion in C/EBPα (C/EBPα KO mice) die shortly after birth due to hypoglycemia, defective hepatic glycogen storage and gluconeogenesis (Linhart et al., 2001). C/EBPα KO mice had no WAT and little BAT; UCP1 mRNA was very low, showing that C/EBPα is essential for the liver and adipose tissue developmental program. Our studies using these mice showed that UCP-1 expression was very low, adipogenesis was impaired and the mitochondria number and function reduced (Carmona et al., 2002). The expression of PGC-1 and thyroid hormone receptors were delayed. BAT D2 activity and BAT T3 were very low indicating that C/EBPα is critical for a correct BAT thyroidal status. It seems that BAT D2 is crucial for the differentiation and activity of fetal BAT and possibly T3 is absolutely necessary for BAT function.

Neonatal hypothyroidism decreases C/EBPα and C/EBPβ expression in liver, but not in BAT (Menendez-Hurtado et al., 1997). In the PEPCK gene C/EBPs and TREs are related, as the activation of C/EBPs is required for a functional TRE (Park et al., 1997).

Besides the C/EBPs, the differentiation of adipocytes is regulated by the PPARs, especially by the PPARγ isoform. PPARs are nuclear receptors acting as transcription factors that regulate gene expression through nutritional stimuli and that control lipid metabolism. Fatty acids, especially arachidonic acid and its metabolites, are natural ligands that activate PPARs. PPARs family has several members: PPARα (activated by fibrate, regulates lipid catabolism), PPARδ and PPARγ, quite specific of adipose tissue. PPARs activate the PPAR response elements (PPRE) present in the promoter of specific target genes, and form heterodimers with the X receptor of retinoic acid (RXR). FAS, aP2, PEPCK, LPL, SCD have PPREs in their promoters. The PPRE sequence of a given gene can bind different isoforms in different tissues, e.g., PPARα in the liver and PPARγ in adipose tissue. PPARs play important roles in adipogenesis, inflammation, atherogenesis, glucose homeostasis and cancer.

During adipogenesis PPARγ is activated (Tontonoz et al., 1995). The ectopic expression of PPARγ induces differentiation into adipocytes upon the stimuli of PPARγ agonists (thiazolidinediones, glitazones) (Tontonoz et al., 1995). PPARγ knockout mice presented several alterations with opposite results: some studies showed a reduced fat formation, and protection against obesity and insulin resistance with lipodystrophy (Jones et al., 2005). The mice with targeted deletion of PPARγ2 have insulin resistance indicating that PPARγ2 is necessary for the maintenance of insulin sensitivity (Medina-Gomez et al., 2005).

The specific coactivator of PPARγ, PGC-1, was identified in 1998 (Puigserver et al., 1998). Under cold exposure PGC-1 increases in BAT. PGC-1 increases the transcriptional activity of PPARγ on the UCP1 promoter and the overexpression of PGC-1 in white adipocytes results in UCP-1 increases as well as mitochondrial enzymes; so PGC-1 is considered a marker of brown adipocytes and activator of BAT (Puigserver et al., 1998). It is also fundamental for hepatic gluconeogenesis, heart function and inflammation (Puigserver and Spiegelman, 2003; Handschin and Spiegelman, 2006; Uldry et al., 2006). Hepatic steatosis, increase in body fat, lower amount of mitochondria, lower respiratory capacity and abnormal cardiac function are found in mice with targeted deletion of PGC1α (Leone et al., 2005).

### T3 regulates gene expression in adipocytes

T3 regulates adipogenesis and the related processes, lipogenesis and lipolysis *in vivo* as well as in cultures of adipocytes (Oppenheimer et al., 1991; Ailhaud et al., 1992). All the isoforms of thyroid hormone receptors TRα-1, TRα-2, and TRβ-1 are present in WAT and BAT and in white and brown adipocytes, and TRα-1 is more abundant (Bianco and Silva, 1988; Teboul and Torresani, 1993; Tuca et al., 1993; Hernandez and Obregon, 1996a). T3 and other hormones regulate the different isoforms. Certain mutations in the TRα gene (P398H mutation) induce increased body fat, visceral adiposity, elevated basal glucose, impaired lipolysis, hyperleptinemia and a reduced adaptive thermogenesis (Liu et al., 2003). This mutation in TRα reduces the binding of PPARα to PPRE elements, interfering with PPARα signaling (Liu et al., 2007).

Many genes expressed during the differentiation program are regulated by T3 and have been extensively studied. The list of genes includes GPD, ME, PEPCK, S14 (Kinlaw et al., 1995), FAS (Moustaid and Sul, 1991), GLUT4, and LPL among many others (Mariash et al., 1980; Blennemann et al., 1995; Bianco et al., 1998). T3 may bind the TREs present in the gene promoters (Petty et al., 1990; Giralt et al., 1991) of those genes. In fact, functional TREs have been identified in several genes as well as its interactions with other nuclear receptors, as PPARs or retinoic acid receptors (Silva and Rabelo, 1997), and also with insulin (IRE) and cAMP response elements (CREs). There is a cross-talk among the regulatory elements (TREs, RAREs, PPARs) of the nuclear receptors as many of them share RXR as pattern of dimerizacion and coactivators, as described in several genes: UCP-1, ME, ACC and others (Mullur et al., 2014). We studied the regulation by T3 of ME and Spot14 (S14) in cultured brown adipocytes (Garcia-Jimenez et al., 1993; Hernandez et al., 1993; Perez-Castillo et al., 1993). ME is a lipogenic enzyme important during differentiation and S14, a T3-target gene, is present in many lipogenic tissues. Both genes increase during differentiation of adipocytes, and are activated by T3. T3 acts at the transcriptional level but also stabilizes the mRNAs produced and T3 effects synergize with insulin. The effect of norepinephrine and retinoic acid was also examined (Hernandez et al., 1993).

The effect of T3 on lipid metabolism also affects other genes as adiponutrin (Adpn, Pnpla3, also called acylglycerol-O-acyltransferase or calcium-independent phospholipase A2 epsilon, iPLA2-epsilon), is a triacylglycerol lipase strongly upregulated by T3 in rat and human white and brown adipocytes (Calvo and Obregon, 2009). Adiponutrin is a lipase with double action as lipase involved the hydrolysis of triacylglycerol and in acyl-CoA transacylation of acyl-glycerols, thereby involved in lipid homeostasis. Other lipases with high impact in BAT are ATGL/desnutrin/Pnpla2 (Ahmadian et al., 2010, 2011) and LPL, that is down regulated by T3 but upregulated when T3+NE is added (Medina-Gomez et al., 2003, 2008).

The fatty acids oxidation is also regulated by T3 in BAT as clearly shown in the D2KO mice (Christoffolete et al., 2004; Castillo et al., 2011) and the FAT-D2KO (Fonseca et al., 2014), with specific deletion of D2 in adipose tissue. D2KO mice show that T3 generated in BAT via D2 is important to accelerate fatty acid oxidation and lipogenesis, as measured by the lipogenic enzymes ACC and ME blunted in BAT. The lack of T3 in D2KO mice impairs thermogenesis and mice survive thanks to compensatory mechanisms in the sympathetic system and increased lipolysis.

**Figure 2:**
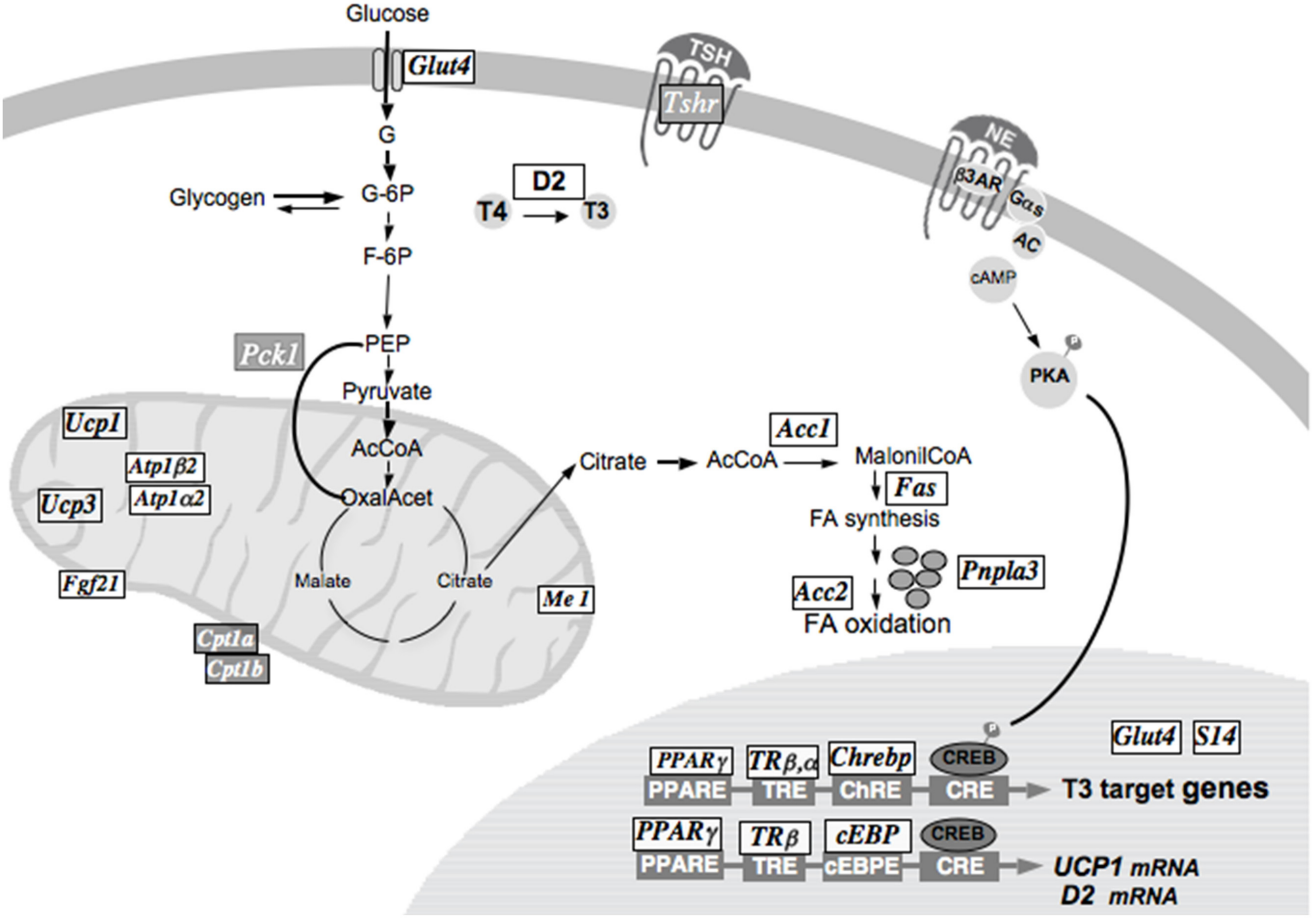
**T3 actions on gene expression in brown adipocytes are represented**. The genes upregulated by T3 are shown in black with white background (UCP1, UCP3, Glut4, Me1, Spot14, Pnpla3, Chrebp). The genes down regulated in the presence of T3 (TSHr, Pck1, Cpt1) are represented in white with a dark background.

Besides its actions on lipid metabolism T3 exert multiple actions on glucose and carbohydrate metabolism, e.g., Glut4 has a TRE in its promoter and the carbohydrate responsive element binding protein (Chrebp) important for Glut4 expression, both are up regulated by T3 in brown adipocytes; other T3 actions include the regulations of gluconeogenesis and glycolysis (Figure 2). T3 also increases ion transport, by increasing several transporters (SLCs) as tramdorin in the plasma membrane as well as mitochondrial transporters, increasing structural genes as keratins (Martinez-De-Mena and Obregon, 2014) and enzymes involved in degradation of metabolites and decreasing TSH receptors.

### T3 actions on thermogenesis

In white adipocytes or 3T3-L1 adipocytes the differentiation process is tested by the increase in lipids, measuring the increase in lipid droplets or increases in lipogenic enzymes. The differentiation in BAT includes an increased thermogenic capacity, measured as UCP-1 increases.

BAT function is the production of heat under cold exposure (facultative thermogenesis). This function is accomplished by the mitochondrial uncoupling protein (UCP-1), which uncouples the oxidative phosphorylation. After activation of the sympathetic nervous system (SNS), NE is released (Ricquier et al., 1986). NE binds to the adrenergic receptors and the adenylyl cyclase is activated increasing cAMP levels; this activates lipolysis, producing FFA which activate UCP1 (Cannon and Nedergaard, 2004). The amount of UCP-1 is the index of the thermogenic capacity of BAT. UCP-1 transcription is activated by NE or cold exposure (Bouillaud et al., 1984; Bianco et al., 1988). T3 increases the adrenergic stimulation of UCP1 (Obregon et al., 1987; Bianco et al., 1988, 1992; Giralt et al., 1990). In thermoneutral conditions and during the intrauterine life, T3 is required for the expression of UCP1 mRNA and euthyroidism is required during the first postnatal days for the increases in UCP1 mRNA (Obregon et al., 1987). In cultured brown adipocytes T3 is required for UCP-1 adrenergic increases, and the stabilization of mRNA transcripts (Hernandez and Obregon, 2000). UCP1 is induced by T3 in fetal rat brown adipocytes in primary culture (Guerra et al., 1996). The effect of T3 on UCP1 is mediated through the TRβ 1 isoform (Ribeiro et al., 2001; Martinez De Mena et al., 2010).

The UCP-1 promoter have CRE sequences (Kozak et al., 1994; Yubero et al., 1998; Rim and Kozak, 2002) in the proximal promoter and an enhancer element containing TRE elements (Cassard-Doulcier et al., 1994; Rabelo et al., 1995), RAREs (Alvarez et al., 1995; Rabelo et al., 1996) and a PPRE in the distal promoter (Teruel et al., 1999). These sequences are promiscuous for its binding to the UCP-1 promoter. Negative regulators of UCP-1 expression are serum and mitogens that activate c-jun (Yubero et al., 1998). Other hormones as glucocorticoids and sexual hormones regulate UCP-1 mRNA.

We studied the effect of Triac, triiodothyroacetic acid, a natural metabolite of T3 produced in the liver, in cultured brown adipocytes (Medina-Gomez et al., 2003). Triac, is a better agonist than T3 for the TRβ isoform; Triac is 10–50 more potent than T3 in stimulating the adrenergic increases of UCP1 and D2, and also down-regulates LPL mRNA in the same fashion. So, Triac is a potent thermogenic agent. The role of Triac was also studied in rats (Medina-Gomez et al., 2008). Triac, in equimolar dosages, was more potent than T3 in rats in the stimulation of UCP-1, LPL, in reducing leptin and low doses of Triac induced ectopic expression of UCP-1 in inguinal WAT (Medina-Gomez et al., 2008), that today are called “beige/brite” adipocytes.

The adrenergic input also increases D2 deiodinase in BAT (Silva and Larsen, 1983), leading to increases in BAT T3. This suggests that T3 has an important role in thermogenesis. Moreover the conversion of T4 to T3 was required for the thermogenic function of BAT (Bianco and Silva, 1987). This is also true for the stimulation of D2 (Hernandez and Obregon, 1996b; Martinez-Demena et al., 2002) that does not occur but in the presence of T3. D2 participates in the formation of BAT, as described above in C/EBPα knockout mice (Carmona et al., 2002), with low UCP1 mRNA, D2 activity, and low mitochondriogenesis. D2 is also implicated in the process of lipogenesis under adrenergic stimuli (Bianco et al., 1998). In D2 knockout mice there is a hyper-adrenergic stimulation compensatory for the lack of T3 production in BAT. Lipogenesis is not providing the FFA levels required during cold exposure, resulting in an impaired adaptive thermogenesis (Christoffolete et al., 2004). Indeed, D2 is a marker of BAT activation (thermogenesis).

Little is known about the role of the deiodinases in white adipocytes. It is evident that they have a role in lipogenesis and in the expression of genes involved in the differentiation program. D1 was found in WAT (Leonard et al., 1983) and human WAT (Ortega et al., 2012) and both, D1 and D2 are found in rat WAT as measured by activity and mRNA (Calvo and Obregon, 2011) and in human preadipocytes (Nomura et al., 2011), but its role has not been established. It remains to be seen if D2 and D1 have different roles from those described in brown adipocytes.

### White, brown adipocytes and “Brite” adipocytes. a role for BAT in humans

Studies comparing brown and white adipocytes in primary culture (Nechad et al., 1983) established that precursor cells from WAT (epididymal fat) and from BAT differentiate into white and brown adipocytes, respectively, with different phenotypes and regulation. The work done during 30 years using primary cultures of precursor cells confirms that precursor cells in each of these tissues are already committed to become brown or white adipocytes. Nowadays it is clear that BAT and WAT derive from different precursor cells. Using microarrays to study both preadipocytes in culture (Timmons et al., 2007), a myogenic signature was found in brown preadipocytes, Myf5, not found in white adipocytes, in which Tcf21 is present, a transcription factor that inhibits myogenesis. Some genes are specific of brown adipocytes or are only found only in white adipocytes.

The lineage of both adipocytes shows that they have a different embryological origin. Brown adipocytes have a myogenic origin defined by the expression of Myf5+, myogenic marker also found in myoblasts (Gesta et al., 2007; Timmons et al., 2007). PRDM16 controls the switch from skeletal muscle to BAT (Seale et al., 2008) activating BAT phenotype (Seale et al., 2007) as well as several markers of BAT as UCP1, D2 and PGC1α. Several genes have been identified that indicate the presence of brown adipocytes such as Myf5, PRDM16, BMP7, BMP4, and Zic1, while others, as Tcf21 is a marker of white adipocytes (Figure 1).

The “beige/brite” adipocytes are found in certain locations of WAT. They are multilocular and express UCP-1. They are found in small amounts and more frequently in certain anatomical locations, e.g., the inguinal fat in rodents. “Brite” adipocytes seem to come from different embryonic precursors than brown adipocytes and express distinct gene signatures (Petrovic et al., 2010), mainly CD137, TBX1, TMEM26 (Walden et al., 2012; Wu et al., 2012) while PRDM16 and PGC1α are markers of “browning.” Its presence, abundance and increase in activity are regulated different than brown adipocytes (Macotela et al., 2012; Walden et al., 2012).

A recent review (Harms and Seale, 2013) lists several experimental models, including knockout mice, in which leaner mice have more active BAT or “brite” adipocytes induced, while a low BAT function is associated to increases in body fat or insulin resistance. When BAT activity increases mice are resistant to weight gain meaning that by increasing BAT activity a reduction in metabolic diseases can be achieved.

Many groups have tried to identify the processes that induce the transition from WAT to “brite” adipocytes and weather BAT and “brite” adipocytes are the same or different cells and the mechanisms of reactivation or induction of BAT activity (Giralt and Villarroya, 2013). The conversion into “brite” adipocytes can be followed using CD137 as a marker of “browning” in human adipocytes (Elsen et al., 2014).

Nowadays the activation of BAT or the “Browning” of WAT is considered a possible strategy to fight obesity. Under extreme cold exposure conditions, a reactivation of BAT adipocytes in inguinal WAT was observed and this was called “convertible” adipose tissue (Loncar, 1991). In this sense, many attempts have been done and there is a number of models in which “browning” of WAT is found (Harms and Seale, 2013), indicating that many signals are able to activate brown fat or to induce “browning” of WAT. Other possible explanation is that a common pathway is activated in all these situations or models, e.g., activation of the adrenergic pathway or also the possible extra-cold experienced by the mice by changes in the fur or skin that augments the cold experienced.

The increases in UCP1 is the golden rule to assess the “browning, as observed using some drugs and also in mice with targeted deletion of a certain genes. Beta 3 adrenergic agonists induce UCP1 in muscle and this provides a mechanism against weight gain (Almind et al., 2007). Brown adipocytes were also found in WAT (Guerra et al., 1998; Xue et al., 2005, 2007). The same effect is observed using models of hyperleptinemia (Commins et al., 1999; Orci et al., 2004) or tungstate (Claret et al., 2005) and we observed that low doses of Triac induce UCP-1 expression in rat inguinal WAT (Medina-Gomez et al., 2008). In mice with targeted deletion of the co-repressor RIP140 (Leonardsson et al., 2004), increases in UCP1 were observed: the mice were lean and resistant to diet-induced obesity. An increasing number of reports find leaner phenotypes associated to increased BAT activity or the presence and activation of “brite” adipocytes, e.g., overexpression of UCP1 in WAT (Kopecky et al., 1995, 1996). More recently the overexpression of Pten results in a phenotype with high UCP1, uncovering the role of Pten promoting energy expenditure (Ortega-Molina et al., 2012). Therefore, when leaner phenotypes are observed in mice, BAT activation or “browning” of WAT should be searched.

#### Human BAT

Due to the implication of BAT in the maintenance of energy balance it has been a growing interest in its possible role in humans, as the reactivation of BAT activity in humans will provide therapeutic tools to fight obesity. BAT was always considered to have minimal importance in humans, though the presence of human BAT was fully accepted under certain conditions: in newborns (Houstek et al., 1993; Krief et al., 1993), patients with pheochromocytoma or outdoors workers or even at all ages (Tanuma et al., 1975, 1976; Huttunen et al., 1981; Zingaretti et al., 2009). There was a growing evidence for the presence of active BAT in humans, as identified using [18F]-fluorodeoxy-glucose-based positron emission tomography (PET) for diagnostic purposes (Nedergaard et al., 2007). In 2009, three papers were published in the NEJM (Cypess et al., 2009; Van Marken Lichtenbelt et al., 2009; Virtanen et al., 2009) where BAT was unequivocally identified and analyzed in humans, including analysis of genes markers of BAT. During the last 5 years it has been a surge of studies on the possible function of BAT in humans and its regulation by cold, diet, obesity and drugs (Zingaretti et al., 2009; Vijgen et al., 2012, 2013; Jespersen et al., 2013; Van Der Lans et al., 2013; Borga et al., 2014; Broeders et al., 2014; Chechi et al., 2014). Several possibilities have been explored as the induction of brite adipocytes in human adipocytes using BMP7 (Pisani et al., 2011). Besides cold, thyroid hormones also regulate human BAT as hyperthyroidism increases BAT metabolism in humans with higher glucose uptake and higher lipid oxidation rate (Lahesmaa et al., 2014).

## Conclusions

In summary, adipogenesis is a complex process that involves a sequential activation of many genes and enzymes, in a cascade of events regulated by transcription factors (C/EBPs, PPARs, PGC1a) that govern the differentiation of adipocytes. T3 regulates many of the enzymes involved in the process of adipogenesis, either directly through the interaction of its nuclear receptors (TRs) with TREs or through the interaction with other nuclear receptors as PPARs or coactivators. The deiodinases, especially D2, play a crucial role producing the T3 required or limiting its levels. D3 increases during proliferation, while D2 plays a crucial role in adipogenesis, thermogenesis and lipid metabolism. The number of genes regulated by T3 in adipocytes continues to grow, not only for lipid metabolism and carbohydrates but also regulating other process unknown to be important for the biology of the adipocyte. Several genes have been identified as markers of brown, white and “beige/brite” adipocytes, establishing that they are distinct cells. “Browning” occurs in some WAT depots under specific conditions or drugs and specific markers of “beige/brite” adipocytes have been identified. Additionally, BAT has been identified in humans and its presence and regulation is being actively studied. The reactivation of BAT and the induction of beige/“brite” adipocytes in humans could represent a therapeutic option to fight obesity.

### Conflict of interest statement

The author declares that the research was conducted in the absence of any commercial or financial relationships that could be construed as a potential conflict of interest.

## Abbreviations

ACC: acetyl-CoA carboxylase
AKT or PKB: protein kinase B
aP2: fatty-acid binding protein
BAT: brown adipose tissue
BMP7, BMP4: Bone morphogenetic protein 7 or 4
cAMP: cyclic AMP
c/EBPα, β, δ: CAAT/enhancer binding protein α, β, δ
CD137: Tumor necrosis factor receptor superfamily 9
ChREBP: carbohydrate responsive element binding protein
Cidea: Cell death activator CIDE-A
D1, dio1: deiodinase 1
D2, dio2: type 2 deiodinase
D3, dio3: deiodinase 3
ERK: Extracellular-signal related kinase
FAS: fatty acid synthase
FFA: Free fatty acid
aFGF and bFGF: acidic and basic fibroblast growth factor
FGF10: Fibroblast growth factor 10
Glut4: Glucose transporter 4
GPD: glycerophosphate dehydrogenase
IBMX: 3-Isobutyl-1-methylxantina
IGFI: Insulin growth factor I
IGFBP: Insulin growth factor binding proteins
LDH: lactic dehydrogenase
LPL: Lipoprotein lipase
MCSF: Macrophage colony-stimulating factor
ME: malic enzyme
Myf5: myogenic factor 5
NE: norepinephrine
PEPCK: phosphoenolpyruvate carboxykinase
PET: positron emission tomography
PGC1α: PPARγ coactivator 1α
PI3K: phosphoinositol-3-kinase
PKA: protein kinase A
PPARγ: Peroxidase proliferator-activated receptor gamma
PPARE: Peroxidase proliferator-activated response element
PRDM16: PR domain containing 16
Pref1: preadipocyte factor 1
Pten: phosphatase and tensin homolog
RARE: retinoic response element
RIP140: receptor-interacting protein 140
SC: subcutaneous white adipose tissue
SCD: stearoyl-CoA desaturase
SLC: Solute carrier transporter
SNS: sympathetic nervous system
Spot14, spot14 or Trsp: Thyroid responsive protein
SVF: stromal vascular fraction
T3: triiodothyronine
T4: thyroxine
TBX1: T-box transcription factor 1
Tcf21: transcription factor 21
Tmem26: Transmembrane protein 26
TNFα: Tumor necrosis factor α
TRα, TRβ: Thyroid hormone receptor α, β
TRE: Thyroid Response Element
Triac: triiodothyroacetic acid
TSH: Thyrotropin
TSHr: TSH receptor
UCP1: uncoupling protein 1
V: visceral white adipose tissue
WAT: white adipose tissue
